# Wanted, a Physician

**Published:** 1889-11

**Authors:** 


WANTED: A physician, a single man with some experience and ability, who is willing to work in a good practice in Texas.

Address

DOCTOR,

LOCK BOX 2, SIPE SPRINGS, TEX.

